# Incidence and Predictors of Mortality among Preterm Neonates Admitted to the Neonatal Intensive Care Unit at Debre Markos Referral Hospital, Northwest Ethiopia

**DOI:** 10.4314/ejhs.v31i5.4

**Published:** 2021-09

**Authors:** Ermias Abebaw, Alemayehu Reta, Getiye Dejenu Kibret, Fasil Wagnew

**Affiliations:** 1 Department of Pediatrics, Debre Markos University, Debre Markos, Ethiopia; 2 College of Health Sciences, Debre Markos University, Debre Markos, Ethiopia

**Keywords:** Preterm, mortality, survival, incidence

## Abstract

**Background:**

Prematurity is the most frequent cause of neonatal death and the second leading cause of under-five mortality. Preterm related complications accounts for 35 % of neonatal deaths within the first week after birth. So far, most studies done in Ethiopia have focused on estimating the prevalence and determinant factors of premature neonatal death. The current study aimed to assess the incidence and predictors of mortality among preterm neonates admitted to neonatal intensive care unit at Debre Markos Referral Hospital.

**Methods:**

An institution-based retrospective follow up study was conducted among premature neonates admitted to Neonatal Intensive Care Unit at Debre Markos Referral Hospital from July 2019 to October 2019. Around 498 patients were selected randomly. A multivariable cox proportional hazards model was fitted to identify predictors of mortality.

**Results:**

A total of 498 preterm babies were followed, and the mean age for follow up at the time of admission to NICU was 15 hours ± 38 SD. Death rate in preterm was estimated to be 27.11% (95% CI: 23.3%, 31.1%). Preterm neonates with gestational age of less than 32 weeks (AHR=1.51; 95% CI: 1.02, 2.24), respiratory distress syndrome (AHR=1.49; 95% CI: 1.03, 2.17), perinatal asphyxia (AHR=1.74, 95% CI: 1.01, 2.76) and congenital malformation (AHR=3.38, 95% CI: 1.21, 8.77) were statistically significant predictors of mortality among preterms.

**Conclusion:**

The incidence of death in preterm neonates is relatively low. Gestational age less than 32 weeks, perinatal asphyxia, respiratory distress syndrome and congenital malformation were found as predictors.

## Introduction

Preterm birth (PTB) refers to the birth of a baby before 37 complete weeks of gestation. It can be further sub-classified based on gestational age as extremely preterm (<28 weeks), very preterm (28 to <32 weeks), moderate to late preterm (32 to <37 weeks) (1).

Infection (36%), preterm birth (28%) and birth asphyxia (23%) are the reported cause of neonatal mortality worldwide (2). Preterm mortality is a significant public health problem throughout the world in general and more predominantly affect low-income countries (3).

Globally, prematurity is the primary cause of death in under-five children. An estimated fifteen million babies, that is more than 1 in 10 babies, born preterm annually (1). Approximately 3 in 4 neonatal deaths of all neonatal deaths were due to preterm birth sequels within the first week of life (2).

About 1.1 million (35%) babies die as a result of preterm birth-related complications. Of which more than 60% of preterm births occur in Africa (38%) and South Asia (39%). In South Asia, preterm birth complications that contribute to under-5 deaths have increased from 16.7% in 2000 to 25.3% in 2015 (5). A meta-analysis done in East Africa revealed that 52% of neonatal deaths were due to prematurity or small for gestational age babies (6).

According to United Nations international child education and fund (UNICEF) and 2016 Ethiopian Demographic and Health Survey (EDHS), and one in twenty one and one in fifteen babies born die before celebrating its first and fifth birthday respectively and preterm birth related complications contributes to the major cause of under-five mortality (2,3). Another report from Federal Ministry of Health (FMOH) showed, prematurity is first cause of neonatal mortality and the fourth cause of under-five mortality in Ethiopia (7).

A study done at Debre Tabor and Addis Ababa showed preterm survival was 68.8% and 70.3 % respectively (5). An institutional-based study in Addis Ababa also revealed 29.7% of neonates died during the follow-up period with an incidence rate of 39.1 per 1000-person day and overall median survival time of 21 days (7). Studies have shown that multiple factors including maternal health factors, obstetric factors and neonatal related factors contribute to neonatal mortality. Maternal health factors include being in rural residency, maternal age less than 20 and greater that 35 and place of birth (8).

Included in the Obstetric factors are Ante natal care (ANC), primipara, pregnancy complications, labor and delivery complications, previous bad obstetric history and multiple pregnancies (2).

Neonatal related risk factors such as male sex, low birth weight, gestational age and neonatal congenital malformations, presence of neonatal clinical problems such as, respiratory distress syndrome (RDS), perinatal asphyxia (PNA), jaundice, hypoglycemia, hypothermia and neonatal sepsis, timely initiation of breastfeeding upon birth and kangaroo mother care (KMC) were found to be significant predictors of time to death for preterm neonates (2).

Despite the international and local efforts, death and cause of preterm births haven't yet reduced as expected. Therefore, this study aimed to assess the predictors and survival status of premature neonate admitted at Neonatal Intensive Care Unit (NICU), Debre Markos Referral Hospital (DMRH).

## Methods

**Study design, period and population:** An institution-based retrospective follow up study was employed at DMRH. The hospital serves as a referral hospital in East Gojjam zone and has been providing service for 3.5 to 5 million populations in its catchment area of Amhara region. The NICU has a total of 18 neonatal beds, and approximately 1218 neonates were admitted per year and 102 admissions per month. The source population was all premature birth neonates admitted to NICU at DMRH. The study population were all premature birth neonates admitted at NICU from January 2017 to December 2018 at DMRH and whose records were available.

The inclusion criteria were all premature birth neonates that were admitted at NICU from January 2017 to December 2018 at DMRH while the exclusion criteria were all Preterm birth neonates' with an incomplete chart records.

**Sample size and sampling technique**: The sample size was computed by using STATA (version 14) considering the following statistical assumptions; two-sided significance level (α) of 5%, power 80 %, Z_a/2_= Z value at 95 % confidence interval =1.96, death rate =78% (7) and Hazard Ratio (HR)=1.54. Simple random sampling technique was used and 498 preterm neonates admitted to NICU from January 2017 to December 2018 were selected and followed for the 28 day postnatal period.

**Data collection procedure**: The available information on the patient charts was first observed, and an appropriate data extraction checklist, which was adapted and modified from different literature, was prepared in English. The checklist comprised socio-demographic characteristics of both premature neonate and the mother, maternal medical disorders and obstetric factors, common medical disorders in the neonate, date of admission and discharge, and outcome of the premature birth. All charts of premature neonates diagnosed from January 1st 2017 to December 2018 at DMRH were reviewed from neonatal registries and recruited in accordance with eligibility criteria.

**Data processing and analysis**: The collected data were coded, entered, cleaned in Epi data version 3.1 and STATA version 14 was used for analysis. Descriptive and inferential statistics were utilized to present the data. Descriptive statistics like frequency and percentage were used to summarize the socio-demographic characteristics of the study participants. And inferential statistics like the Hazard ratio was computed to determine the association between the dependent variable and different independent variables. Kaplan Meier was used to estimate survival time, to depict the pattern of death and to compare the survival curves among different categories. The log-rank test was also used to look at statistical differences between categories of variables. Bivariable analysis of the individual variables with preterm neonatal survival was carried out to determine the possible significant explanatory variables to be included in the Cox-proportional hazard model. Variables significant at a p-value < 0.25 in the bivariable analyses were eligible to enter into the final multivariable analysis to identify predictors of time to death. The Cox proportional hazard assumption was checked using graphical approaches (Log-log plot) and the Schoenfeld residual test. Hazard ratio (HR) with 95% confidence interval was calculated for the significant predictive variables, and a statistical significance was declared at (P< 0.05). The goodness of fit of the final model was assessed by Cox-Snell residuals with the Nelson Aalen cumulative hazard function graph.

**For this study the following operational definitions were used.**

**Censored:** premature neonate at NICU that will still be alive at the end of the study or lost to follow up including discharged to home, discharged against medical advice or transfer out to other health institutions without knowing the outcome.

**Follow up time**: The time starting from admission until either an event or censorship occurs. **Medical disorders in mother**: Any history of medical diagnosis in the mother as it has been registered on the neonate's medical record.

**Medical disorders in the neonate**: Any recorded medical diagnosis for the premature neonates on their medical records.

**Survival status**: Is the final outcome of the premature neonate, either death or censored.

**Survival time**: Measures the follow-up time from a defined starting point/from admission in NICU up to the occurrence of the outcome/last neonatal period.

**Time origin**: Admission of the premature neonate at NICU.

**Time scale**: Days from the admission of a premature neonate.

**Ethical consideration**: Ethical clearance and approval to conduct this research was obtained from the Research and Ethical Review Committee of College of Health Science, Debre Markos University. Permission to conduct the study was requested from the East Gojjam zone Health Department. Prior to administering the questionnaire, the aims and objectives of the study were clearly explained to the hospital manager and then written informed agreement was obtained from Debre Markos Referral Hospital. This study didn't expose premature neonates to unnecessary risk as a result of reviewing their medical records. To keep confidentiality, each collected data were coded and locked in a separate room prior to enter into the computer. Following entry into the computer, all data were protected by password. Names and unique medical registered numbers (MRN) were not included in the data collection set-up.

## Results

**Socio-demographic characteristics of the mother and neonate**: About 258 (51.81%) of the study subjects were males, and the majority of them 433 (86.95%) came from rural area. The mean age of mothers were found to be 27+5.53 SD years old, and 357 (71.69%) were in the age category between 20 to 34 years. In addition, nearly three-fourths, 263 (72.45 %), of premature neonatal death was concentrated in this age groups of the mother. The mean age for the cohort at the time of admission to NICU was 15 hours ± 38 SD. The mean weight was also found to be 1743.51 ±397.83 SD grams.

**Maternal and pregnancy Characteristics**: Among the total mothers enrolled in the study, 486 (97.59%) had ANC follow up. Of the 135 died, preterm born to para two and less Mother's accounts for the highest number of premature death 76 (56.3%). The result of this study also indicated that 126 (25.3%) of the mother were diagnosed to have maternal complications like preeclampsia 52 (41.27%), premature rupture of membrane (PROM) 45 (35.71%), Abruptio placenta 28 (22.22%) and 4.76% experienced other medical complications. Among 28 (5.62%) of the mothers who were diagnosed to have medical problems 14 (50%) had HIV/AIDs, 1 (3.57%) had hypertension and 1 (3.57%) had anemia. Moreover, we found that 413 (82.93%) of mothers gave birth through spontaneous vaginal delivery, 77 (15.46%) of them with caesarian section (CS) and 8 of them (1.61%) with instrumental delivery. Of those who delivered via cesarean section (CS), spontaneous vaginal delivery (SVD) and instrumental delivery 12 (8.89%), 119 (88.15 %) and 4 (2.96%) neonates died during follow-up period respectively (Table 1).

**Table 1:** Maternal and pregnancy related characteristics of the study participant that was admitted to NICU of DMRH, Debre Markos, Ethiopia, January 2017 to December 2018 (n=498)

Covariates	Category	Number & Total (%)	Status	

			Death (%)	Censored (%)
Parity	>2	222(44.58%)	59(43.7%)	163(44.9%)
	<2	276(55.42%)	76(56.3%)	200(55.10%)
ANC	Yes	486(97.59%)	133(98.52%)	353(97.25%)
	No	12(2.41%)	2(1.48%)	10(2.75%)
Multiple Gestation	Yes	176(35.34%)	48(35.56%)	128(35.26%)
	No	322(64.66%)	87(64.44%)	235(64.74%)
Maternal complication	Yes	126(25.3%)	33(24.44%)	93(25.62%)
	No	372(74.7%)	102(75.56%)	270(74.38%)
PROM	Yes	45(9.04%)	11(8.11%)	34(9.37%)
	No	453(90.96%)	124(91.85%)	329(90.63%)
Preeclampsia	Yes	52(10.44%)	11(8.15%)	41(11.29%)
	No	446(89.56%)	124(91.85%)	322(88.71%)
Abruptio placenta	Yes	28(5.62%)	12(8.89%)	16(4.41%)
	No	470(94.38%)	123(91.11%)	347(95.59%)
Mode of delivery	Spontaneous vaginal delivery	413(82.93%)	119(88.15%)	294(80.99%)
	Caesarian section	77(15.46%)	12(8.89%)	65(17.91%)
	Instrumental	8(1.61%)	4(2.96%)	4(1.10%)
HIV/ADIS	Yes	14(2.81%)	5(3.7%)	9(2.48%)
	No	484(97.19%)	130(96.3%)	354(97.52%)
Anemia	Yes	1(0.2%)	0(0%)	1(0.28%)
	No	27(99.8%)	135(100%)	362(99.72%)
Hypertension	Yes	1(0.2%)	0(0%)	1(0.28%)
	
	No	497(99.8%)	135(100%)	362(99.72%)

**Neonatal characteristics**: Almost three fourths 386 (77.51%) of neonates were with gestational age of greater than or equal to thirty-two weeks. The proportion of very low birth weight accounted for 347 (69.68 %). More than half, 262 (52.72%) of preterm neonates were hypothermic during admission time. Almost all, 496 (99.6 %) of preterm neonates had neonatal complications during admissions such as sepsis 423 (85.11%), respiratory distress syndrome 185 (37.22%), perinatal Asphyxia 69(13.88%,), congenital malformation 10 (2.02%) and jaundice 37 (7.44%) (Table 2).

**Table 2:** Common medical diagnosis of premature neonate that was admitted to NICU of DMRH, Debre Markos, Ethiopia, January 2017 to December 2018 (n=498)

Covariant	Category	Number & Total (%)	Status	

			Death (%)	Censored (%)
Neonatal complication	Yes	496(99.6%)	135(100%)	361(99.45%)
	No	2(0.40%)	0(0%)	2(0.55%)
RDS	Yes	185(37.15%)	72(53.33%)	113(31.13%)
	No	313(62.85%)	63(46.67%)	250(68.87%)
Sepsis	Yes	423 (84.94%)	113(83.7%)	310(85.4%)
	No	75(15.06%)	22(16.3%)	53(14.6%)
PNA	Yes	69(13.86%)	27(20%)	42(11.57%)
	No	429(86.14%)	108(80%)	321(88.43%)
Hypothermia	Yes	262(52.61%)	73(54.07%)	189(52.07%)
	No	236(47.39%)	62(45.93%)	174(47.93%)
Congenital malformation	Yes	10(2.01%)	5(3.7%)	5(1.38%)
	No	488(97.99%)	130(96.3%)	358(98.62%)
Jaundice	Yes	37(7.43%)	9(6.67%)	28(7.71%)
	No	461(92.57%)	126(93.33%)	335(92.29%)
Other	Yes	39(7.83%)	17(12.59%)	22(6.06%)
	
	No	459(92.17%)	118(87.41%)	341(93.94%)

**Figure 1:**
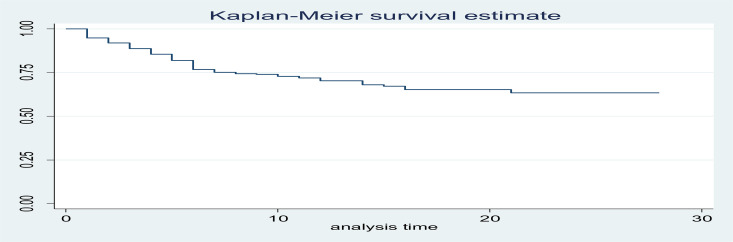
Summary of Kaplan-Meier estimate of survival probability among preterm neonate admitted at Debre Markos Referral Hospital NICU, Debre Markos, Ethiopia, January 2017 to December 2018. (n=498)

**Preterm survival rate**: Four hundred ninety-eight participants were followed for different time ranging from one day to 28 days. The mean survival time was 9.28 days. This finding showed that overall preterm survival proportion was 363 (72.89 %) (95% CI: 68.7%, 76.7%). The total extent of follow-up was 4625 person-day, with an incidence rate of 29.2 deaths per 1000 person-day observation (95% CI: 24.7, 35). (Figure 1).

**Figure 2:**
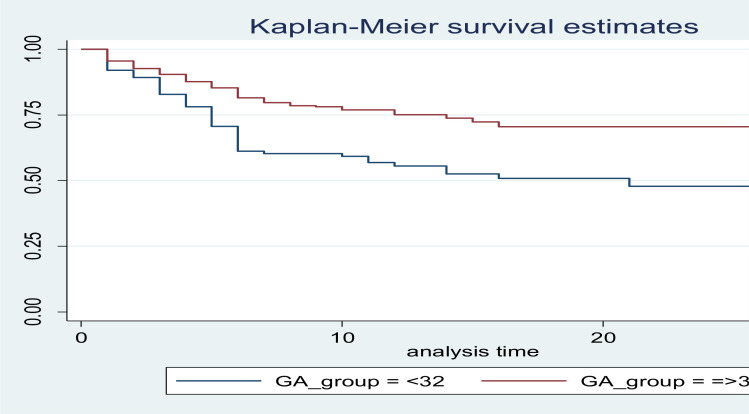
Kaplan-Meier estimate of survival probability among preterm neonate with categories of gestational age admitted at Debre Markos Referral Hospital NICU, Debre Markos, Ethiopia

**Predictors of preterm survival**: In multivariable Cox regression analysis, gestational age, respiratory distress syndrome, perinatal asphyxia and congenital malformation were significant predictors. The result of the multivariable analysis showed that preterm neonates with gestational age less than 32 weeks were 1.5 times more likely to die as compared to those neonates born after 32 weeks of gestation (AHR: 1.51 (1.02, 2.24)) (Table 3) (Figure 2). Premature neonate with respiratory distress syndrome at the base line were also 1.49 times more likely to die compared to those neonates without RDS (AHR: 1.49 (1.03, 2.17)) (Table 3).

**Table 3:** Cox-proportional hazard model of predictors of Preterm survival among preterm neonate admitted at DMRH NICU, Debre Markos, Ethiopia, January 2017 to December 2018 (n=498)

Predictor	Category	CHR (95% CI)	AHR (95% CI)
Sex	Male	1.13(0.80,1.58)	1.13(0.71,1.51)
	Female	1	1
Residence	Rural	0.89(0.55,1.45)	0.9(0.55,1.47)
	Urban	1	1
Gestational Age	<32	2.02(1.42, 2.86)	1.51(1.02, 2.24)**
	>32	1	1
Abreptio placenta	Yes	1.61(0.89,2.9)	1.39(0.76, 2.53)
	No	1	1
Neonatal age	<24hr	1.97(0.92, 4.23)	1.75(0.80,3.83)
	>24hr	1	1
Birth weight	1000–1499	3.05(0.95, 9.78)	2.46(0.73,8.32)
	1500–2499	0.53 (0.37, 0.75)	1.51(0.47,4.89)
	2500–3999	1	1
RDS	Yes	1.98(1.41, 2.78)	1.49(1.03, 2.17)**
	No	1	1
Sepsis	Yes	0.63(0.310, 0.99)	0.78(0.47, 1.29)
	No	1	1
PNA	Yes	1.70(1.12,2.59)	1.74(1.10, 2.76)**
	No	1	1
Congenital malformation	Yes	2.26(0.92, 5.53)	3.38(1.210,8.77)**

	No	1	

** Significant predictors in the multivariable analysis at P<0.05; *Crude*

HRs reflect bivariate modeling results, whereas adjusted HRs are taken from a multivariate model containing all variables in the table and categories labeled as 1 are considered as reference categories.

Premature neonates who had perinatal asphyxia at the time of admission were 1.74 times more likely to die than those neonates without perinatal asphyxia (AHR: 1.74 (1.01, 2.76)).

**Figure 3:**
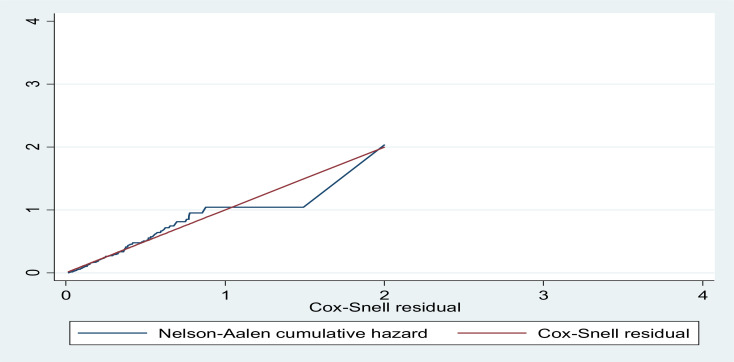
Nelson-Aelen cumulative hazard for the final model on survival probability among preterm neonate admitted at Debre Markos Referral Hospital NICU, Debre Markos, Ethiopia, January 2017 to December 2018 (n=49)

Neonates with congenital malformation had 3.4 times more likely to die as compared to their counterparts (AHR: 3.38 (1.21, 8.77). It is shown that the hazard function follows the forty-five degree line very closely except for very large values of the time. It is very common for models with censored data to have some wiggling at large values of time. Therefore, it is presumed that the final model fits the data well (Figure 3).

## Discussion

Our study aimed to determine incidence of preterm mortality and its predictors that were admitted to NICU at DMRH. Based on this study finding the overall mortality of premature neonates admitted to NICU in DMRH during the study period was 135 (27.11%) (95% CI: 23.3%, 31.1%). The overall mortality rate of this study was consistent with studies conducted in Nigeria (27.69%) (9), Johannesburg 26.5% (10) and Iran 27.4% (11).

This finding is also consistent with a study done in University of Gondar, Ethiopia, which is 25.2%, Tikur Anbessa specialized hospital in Addis Ababa (29.77%) and Debre Tabor general hospital (31.2%) but the result is lower than a study done in Jimma university hospital (34.9%) (2, 5, 7, 12). This possible source of variation in mortality rate might be difference in sample size, methodology and study population which selectively take preterm as our study subject. Other possible explanation might be the difference in the study period as there were changes in treatment modality.

At the end of follow up, the overall incidence of mortality was found to be 29.2 deaths per 1000 person-day observation (95%CI: 24.7, 35). This finding is lower than a study conducted in Jordan which is 123 per 1000 live births (13), in Tigray region, Ethiopia, which is 42 per 1000 live birth (14) and Tikur Anbessa Hospital which is 39.1 per 1000 person day observation (7). This marked difference might be attributed to difference in sample size, study period and area, and the characteristics of the study participants.

This study showed that preterm neonates who were born with a gestational age of less than 32 weeks are more likely to die than neonate born with a gestational age of greater than or equal to 32 weeks. The result of this study is consistent with a study done in Tikur Anbessa hospital, Debre Tabor and Gondar (2,5,7). Similarly, it was shown to have a consistent result with a study done in Rwanda and Iran (11, 15). This shows that low gestational age is associated with low birth weight and neonate would be at higher risk of death because of prematurity related complications with low gestational age.

Likewise, preterm neonates with RDS had more hazard of death than preterm neonates without RDS. This finding is consistent with a study done in Jimma, Debre Tabor and Gondar.(2,5,12,16).

This might be due to inadequate perinatal care attributed to less use of surfactant, inadequate availability of noninvasive and invasive ventilation methods and concomitant overlooked complications of RDS that increase the risk of mortality in preterm neonate.

In addition, neonates with PNA had a higher risk of mortality as compared with their counterparts. Consistent results have been found in other studies (2,7,16,17). The possible reasons for this could be PNA leads to progressive hypoxemia and hypercapnia resulting in central nervous and other end organ damage. The presence of neonatal encephalopathy is considered as an essential etiologic link that predicts mortality or severe disability.

Furthermore, preterm neonates diagnosed with congenital malformation had a higher risk of death than their counterparts. This finding is in consistent with studies done in Addis Ababa, Tikur Anbessa Specialized Hospital and Iran (11,12,18). These could be due to congenital anomalies affect multi-organ system, and the risk of mortality is hence attributed to various systemic complications (19).

In conclusion, the incidence of death in preterm neonate is low compared with study findings in similar set ups. Survival of preterm neonate was lower for gestational age less than 32 weeks, perinatal asphyxia, Respiratory distress syndrome and congenital malformation.

Since the data were collected from secondary source; some important predictors such as gravidity, early initiation of breast feeding, APGAR score at 1st and 5th minutes and some maternal medical problems like anemia and diabetes were missed which are a significant predictor of mortality in premature babies. Moreover, the study area covers only DMRH that limits generalizability to all hospitals in Amhara region and Ethiopia. Selection bias is possibly introduced during secondary data collection because patients with incomplete records were excluded so that the incidence of death may be underestimated.

Preterm mortality following common preterm related complications like RDS, PNA is one of the highest in our hospital and hence DMRH should facilitate more research to find out the root causes of predictors of premature neonatal death and ascribed maternal adverse outcome. Health care provider and hospital management should also strengthen careful follow up and regular monitoring of patients with early preterm, respiratory distress syndrome, perinatal asphyxia and congenital malformation which are significant predictors of mortality in preterm and standardized care for admitted preterm neonate, including promotion of advanced clinical care has to be strengthened through providing timely training so as to reduce the rate of premature neonatal death.

Furthermore, longitudinal prospective cohort study is strongly recommended to identify additional factors that determine preterm survival and also see the outcomes of those preterm who are censored in this study.
